# Quantification of persistent organic pollutants in breastmilk and estimated infant intake, Norway

**DOI:** 10.1111/mcn.13759

**Published:** 2024-11-05

**Authors:** Kristina R. Nermo, Jan L. Lyche, Gabrielle Haddad‐Weiser, Tonje E. Aarsland, Siri Kaldenbach, Beate Solvik, Anuschka Polder, Tor A. Strand, Kjersti S. Bakken

**Affiliations:** ^1^ Department of Microbiology Innlandet Hospital Trust Lillehammer Norway; ^2^ Center for International Health, University of Bergen Bergen Norway; ^3^ Faculty of Veterinary Medicine, Norwegian University Life Sciences (NMB) Ås Norway; ^4^ Women's Clinic, Innlandet Hospital Trust Lillehammer Norway; ^5^ Department of Clinical Medicine University of Oslo, Faculty of Medicine Oslo Norway; ^6^ Department of Pediatrics Lillehammer Hospital, Innlandet Hospital Trust Lillehammer Norway; ^7^ Department of Research Innlandet Hospital Trust Lillehammer Norway

**Keywords:** breastmilk, brominated flame retardants, infants, organochlorine pesticides, polychlorinated biphenyls

## Abstract

Persistent organic pollutants (POPs) are environmental contaminants that can accumulate in human tissues and pose potential health risks. Despite global efforts to reduce their prevalence, follow‐up studies are needed to see if the measures are successful. Since most infants in Norway are breastfed for the first 6 months of life, monitoring POP contamination in breastmilk is important for children's health and development. This study aims to evaluate the current levels of various POPs in women's breastmilk in Innlandet County, Norway. A cross‐sectional study was conducted measuring concentrations of 35 different POPs, including polychlorinated biphenyls (PCBs), chlordanes (ChlDs), hexachlorocyclohexanes (HCHs), dichlorodiphenyltrichloroethanes (DDTs), Mirex, and brominated flame retardants in 120 breastmilk samples. The study analysed the impact of maternal age, parity, pre‐pregnancy BMI, and infant age on POPs levels and compared the estimated daily intake per body weight of infants to existing health guidelines. The detected percentages for PCBs were 100%, for DDTs 98.3%, and for ChlDs 98.3%. The highest median concentration was found for ΣPCBs (26.9 ng/g lw). Maternal age, parity, and infant age were significant determinants of POP concentrations. Most infants exceeded the health‐based guidance values for ΣPCB, and 6.4% percent did so for ΣHCHs. Despite lower POPs concentrations in breastmilk than in earlier studies, many breastfed infants are still exposed to levels exceeding health‐based guidance values. Although the study's design had limitations, the study provides updated population‐based data on POPs in breastmilk. Continued monitoring and research are necessary to understand and mitigate potential health risks associated with POPs.

## Key messages

- Studying POPs in breast milk and their health impacts.
- Breast milk POPs analysis via GC‐MS.
- Maternal age, parity, and infant age impact POPs concentrations.
- Infants exceed safe limits for Σ_7_PCBs and ΣHCHs.
- Reduced POPs compared to past studies, but high infant exposure remains.

## Graphical abstract

Organochlorine pesticides (OCPs), polychlorinated biphenyls (PCBs), and brominated flame retardants (BFRs) in human milk samples collected in 2020/2021, Norway. Of 110 infants, 99.1% and 6.4% of ingest Ʃ_7_PCBs and ƩHCHs (hexachlorocyclohexanes) above health‐based guidance values through human milk.

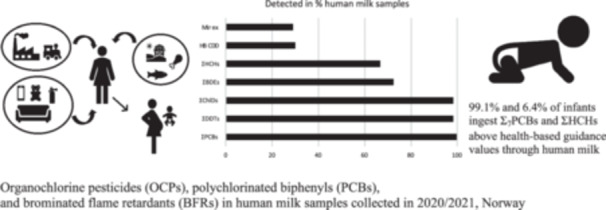

## 1 INTRODUCTION

Persistent organic pollutants (POPs) are known for their extended half‐lives in soils, sediments, air, and biota and are resistant to biological, photolytic, and/or chemical degradation (Ritter et al., 1995). POPs are hydrophobic and lipophilic, and therefore predominantly accumulate in the fat tissue of both humans and animals (Ritter et al., 1995). Due to their toxic properties, they have the potential to negatively impact the environment and human health (Alharbi et al., 2018). In humans, POPs have been associated with effects on the immunological, reproductive, and endocrine systems (Vrijheid et al., 2016), and are linked to increased risk of cancers (Azizi et al., 2023). Contamination along food production chains lead to transfer of POPs into the human diet, with meat, dairy, eggs, and fish being the leading cause of human exposure, accounting for over 90% of human intake (Cok et al., 2009; Polder et al., 2010; Rodríguez‐Hernández et al., 2015).

In 2004, the Stockholm Convention, an international treaty aimed at protecting human health and the environment from POPs, entered into force (SC Stockholm Convention, 2023). Though there has been a ban on POPs for many years, they can still be detected in human specimens such as serum, cord blood, placenta, and breastmilk (Björvang et al., 2021; Odland et al., 2021). Infants and young children may be particularly susceptible to the effects of POPs due to critical phases of growth and development (Damstra, 2002). Children have a limited ability to detoxify these chemicals and less fat tissue to deposit POPs (Damstra, 2002). Polychlorinated biphenyls (PCBs) exposure during pregnancy has been associated with adverse birth outcomes such as smaller head circumference and lower gestational age (Kezios et al., 2012; Ribas‐Fitó et al., 2002; Tan et al., 2009). Similarly, elevated hexachlorobenzene (HCB) levels in maternal and cord blood have been associated with lower gestational age, whereas the relationship between hexachlorocyclohexanes (HCHs) and head circumference and crown‐heel length is less clear (Brucker‐Davis et al., 2010; Eggesbø et al., 2009; Eskenazi et al., 2004; Lopez‐Espinosa et al., 2011; Ribas‐Fitó et al., 2002). HCHs and PCBs exposure has been associated with reduced motor development, language, and cognitive abilities at 18 months of age (Ruel et al., 2019; Wang et al., 2021). Additionally, exposure to PCBs in utero and early‐life has been linked to a decrease in general abilities measured as the intelligence quotient (IQ) and verbal IQ at 11 years of age, as well as an increased risk of attention‐deficit/hyperactivity disorder (ADHD) and autism spectrum disorder (ASD) (Forns et al., 2016; Grova et al., 2019; Jacobson & Jacobson, 1996; Lenters et al., 2019).

For most infants in Norway, maternal breastmilk is the primary food source during the first 6 months of life (Paulsen et al., 2020). Moreover, breastmilk is the primary source of POPs intake in infants (Barr et al., 2006; LaKind et al., 2004). Previous studies from Norway have reported median concentration of 20 PCBs in breastmilk to be from 177 ng/g lipid weight (lw) in 2000–2002 to 103 ng/g lw of 18 PCBs in 2002–2006 (Polder et al., 2008, 2009). The most comprehensive breastmilk study in Norway that we reviewed reported a median concentration of 14 PCBs for 1199 women in the HUMIS cohort (1999–2008) to be 93 ng/g lw (Lenters et al., 2019), indicating a downward temporal trend during this period.

Even though POPs concentrations in breastmilk have been consistently monitored in Norway since 1978, both in national studies and as part of the World Health Organization (WHO) international breastmilk monitoring programme (Skaare, 1981), available data on Norwegian infants' exposure to POPs through breastmilk since 2010 remains limited. The aim of this study is to analyse the concentrations of POPs in breastmilk samples collected from women in Norway during 2020/2021 and to compare their infants' estimated intake levels against the corresponding health‐based guidance values for evidence‐based recommendations regarding breastfeeding.

## 2 MATERIALS AND METHODS

### 2.1 Recruitment and data collection

For this analysis, breastmilk samples from women who were initially part of a cross‐sectional study on iodine nutrition conducted in Innlandet County, Norway were used. A total of 355 mother–child pairs with children up to 2 years of age from healthcare centres spread across 30 municipalities were enlisted from November 2020 to October 2021. These municipalities were randomly chosen from a list of all municipalities in the county. The frequency of a municipality's inclusion ranged from 1 to 10 times, based on the birth rate in 2019. The goal was to enlist four mother‐child pairs from the designated healthcare centre for each time a municipality appeared on the list. Local nurses were trained on participant recruitment protocols and were equipped with the necessary tools. A more thorough description of the recruitment process is elaborated upon elsewhere (Aarsland et al., 2023).

**Figure 1:**
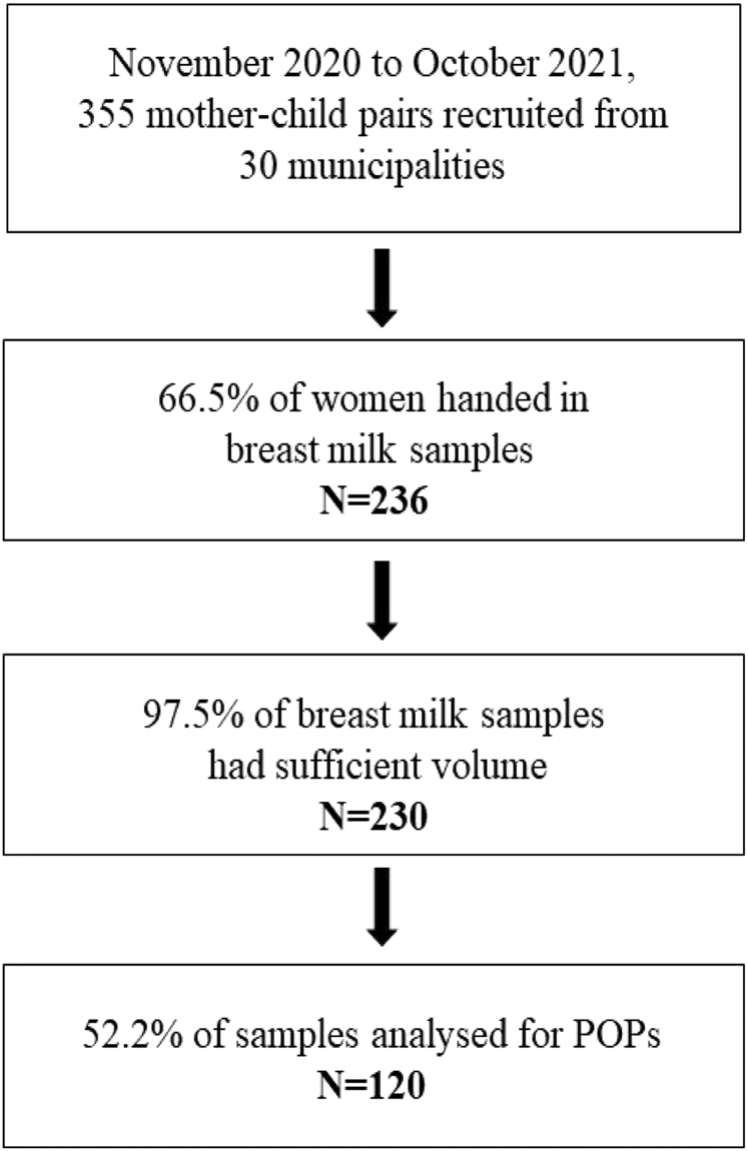
Flowchart of the study population and the collection of breast milk. POP, persistent organic pollutant.

Mothers were given a 50 mL polypropylene (PP) centrifuge tube (Sarstedt) to collect breastmilk and were instructed to store it in the refrigerator before delivering it to the health care center. Upon collection, the breastmilk samples were kept refrigerated at 4°C before being allocated into 5‐ and 10‐mL PP centrifuge tubes (Sarstedt). These tubes were then transferred to long‐term storage at −80°. The samples were kept frozen during transport and until analysis. For the purpose of this study, 120 samples were randomly selected based on the birth rate in the different municipalities included in the iodine study (Figure 1).

### 2.2 Main outcome variables

Each breastmilk sample was measured for fat percentage and the concentration (ng/g wet weight) of 35 different POPs. These comprised 14 organochlorine pesticides, which included α‐, β‐ and γ‐hexachlorocyclohexane (HCHs), oxychlordane, *trans*‐ and *cis*‐chlordane, *trans*‐ and *cis*‐nonachlor (ChlDs), *p,p*′‐dichlorodiphenyldichloroethylene (DDE), *o,p*′‐dichloro‐diphenyldichloroethane (DDD), *p,p*′‐DDD, *o,p*′‐dichlorodiphenyltrichloroethane (DDT), *p,p*′‐DDT (DDTs), and Mirex. Additionally, there were 13 polychlorinated biphenyls (PCBs) identified: PCB‐28, ‐31, ‐101, ‐105, ‐114, ‐118, ‐138, ‐153, ‐156, ‐157, ‐170, ‐180, and ‐189. Lastly, eight brominated flame retardants were analysed: brominated diphenyl ether (BDE)‐28, ‐47, ‐99, ‐100, ‐153, ‐154, ‐183, and hexabromocyclodo‐decane (HBCDD).

### 2.3 Other variables

Participating women were requested to complete a questionnaire with questions about maternal age, height (cm) and pre‐pregnancy weight (kg), country of birth, cigarette smoking (yes, no, or previous), and parity (in numbers), as well as the infant's age (months) and sex.

### 2.4 Extraction and fat determination

Sample preparation and chemical analyses were based on a method first described by Brevik, (1978) and later updated by Polder et al. (2014). Approximately 4 g of breastmilk were added to 80 mL glass centrifuge tubes, followed by the addition of internal standards (PCB‐29, ‐112, and ‐207, BDE‐77, ‐119, and ‐181) and solvents (6% NaCl, water, acetone, and cyclohexane). Samples were extracted, sonicated and centrifuged. The upper phase was collected, and the extraction was repeated with additional acetone and cyclohexane at a 2:3 ratio. The combined upper phases were then concentrated using a Biotage TurboVap II and reconstituted to 5 mL. An aliquot of 1 mL of the sample was used for gravimetric fat determination, and the remaining sample was further prepared for analysis by GC‐MS.

One mL of the sample extraction was transferred to a pre‐weighed 8‐dram glass bottle. The solution was evaporated in a sand bath at 40°C overnight. The glass bottles were then weighed again, and the fat content was determined using the following formula:

$$\text{Fat}\;\%=\frac{\left(\mathrm{End}\;\mathrm{weight}-\text{dramglass weight}\right)\times 5}{\mathrm{Start}\;\;\;\mathrm{weight}\;\;\mathrm{of}\;\;\mathrm{sample}}\times 100.$$

### 2.5 Sample clean‐up

The remaining 4 mL of sample was transferred to 10 mL glass test tubes and mixed with 4–6 mL of 97.5% H_2_SO_4_, depending on the amount of fat in the sample. Samples were kept in the dark at room temperature for 1 h before centrifuging and collection of the upper phase. Samples were then concentrated to a final volume of 0.40 mL using a gentle stream of nitrogen and transferred to amber GC vials.

### 2.6 GC‐MS analyses of organochlorine pesticides (OCPs), PCBs, and brominated flame retardants (BFRs)

Pesticides and PCBs were separated and detected using GC‐MS/ECD as previously described by Mwakalapa et al. (2018) and Polder et al. (2014), on an HRGC (Agilent 6890, Agilent Technologies) with a programmable temperature vaporization (PTV) injector, coupled to an ECD and MS detector (Agilent 5975 C, Agilent Technologies) in negative chemical ionization mode (NCI) with selected ion monitoring (SIM). Analytes were separated on a 60 m DB‐5 MS column (0.25 mm i.d. and 0.25 mm film thickness) (J&W Scientific, Agilent Technologies). The injection volume was 1 μL and the carrier gas was helium at a constant flow of 1.3 mL/min. Brominated flame retardants were separated and detected on an HRGC (Agilent 8890, Agilent Technologies) with a multimode inlet (MMI) injector in pulsed splitless mode, coupled to an MS detector (Agilent 5977) in NCI mode with SIM. Analytes were separated on a 15 m DB‐XLB column (0.25 mm i.d. and 0.10 mm film thickness) (J&W Scientific, Agilent Technologies). The injection volume was 2 μL. The inlet temperature programme had an initial temperature of 115°C followed by a ramp rate of 900°C/min to 320°C for 4 min. Helium served as the carrier gas at a constant flow of 1.2 mL/min. The initial oven temperature was 80°C held for 1.5 min, followed by a ramp rate of 33°C/min up to 320°C for 9 min, and the transfer line to the MS detector was held at 300°C.

All analytes were quantified using a calibration curve containing 6–10 points, and all calculations were performed within the linear range of the curves.

### 2.7 Quality assurance (QA) and quality control (QC)

Chemical analyses were conducted at the Environmental Toxicology Laboratory of the Norwegian University of Life Sciences in Ås, Norway. The laboratory is accredited for testing chemicals in biological samples according to the standards of NS‐EN ISO/IEC 17025 (TEST 137).

Each analytical series contained one blind sample and two spiked recovery samples of cow's milk, one sample of the laboratory's own internal reference standard of harp seal blubber (*Pagophilus groenlandicus*), and three procedural blanks containing only solvents. All glassware used in sample preparation was rinsed with a mixture of cyclohexane and acetone immediately before use to ensure the removal of dust. The three blank samples were treated exactly the same as real samples to account for any potential contamination present in each step of the sample preparation. Any levels of analytes reported in the blanks were subtracted from samples. Results from recovery samples and internal reference standards were approved before analysis of samples and are monitored over time to observe trends in results. During GC runs, one standard was repeated approximately every 10 samples to monitor potential drift in retention times, and each analyte quantified with MS also had one qualifier ion to ensure the correct selection of peaks. As part of the accreditation, the laboratory also participates in annual interlaboratory testing including Quasimeme and AMAP studies, as well as routine analyses of various certified reference materials (CRMs).

Limits of detection (LOD) for individual analytes were defined as three times the noise level of each analyte, while limits of quantification (LOQ) were defined as three times the LOD. The LODs in ng/g (wet weight, ww) for each group of analytes were as follows: β‐HCH = 0.003, α‐HCH, γ‐HCH, Mirex, and all chlordanes = 0.002, *p*,*p*′‐DDE = 0.046, *o*,*p*′‐DDD = 0.023, p,p′‐DDD = 0.021, *o*,*p*′‐DDT = 0.019, *p*,*p*′‐DDT = 0.025, PCB‐28 and ‐31 = 0.004, PCB‐101 = 0.025, PCB‐114 = 0.003, and the remaining PCBs = 0.002, BDE‐28 and ‐154 = 0.006, BDE‐47 = 0.008, BDE‐99 = 0.021, BDE‐100 and ‐153 = 0.009, BDE‐183 = 0.025 and HBCDD = 0.056 (Table 2).

### 2.8 Statistical analyses

The data were analysed using STATA 17.0 software (StataCorp, College Station, TX, 2023). Descriptive data were presented as numbers and percentages. Maternal age and pre‐pregnancy body mass index (BMI, kg/m^2^) were presented as means and standard deviations. Concentrations of the POPs were presented in ng/g ww and lw as median with 5th and 95th percentile. Calculations were performed to show the POPs concentrations in ng/g lw, using each sample's individual fat percentage. Negative binomial regression models were used to explore associations between concentrations of POPs and maternal age, parity, BMI before pregnancy, and infant's age in months. This regression model was chosen based on the Akaike Information Criterion and the Bayesian Information Criterion, which are used to compare the goodness of fit of different statistical models while penalizing for the number of parameters to prevent overfitting. In samples where POPs were not detected, we assigned them a value of half the lowest detectable limit (LOD). Results are shown as ratios of means (RM) with 95% confidence intervals. Lastly, the amount of exposure to POPs for each infant was calculated and these levels were compared to health‐based guidance values. To estimate daily intake per body weight (bw), estimations from Rios‐Leyvraz and Yao (2023) were used, which provides the average daily breastmilk intake per body weight (mL/kg per day) for children up to 17 months of age. Acceptable daily intake (ADI) for sum of HCHs (ƩHCHs) (WHO, 2003) and tolerable daily intake (TDI) for sum of PCBs (ƩPCBs) (Skåre et al., 2008) was set at 10 ng/kg bw, TDI for sum of pesticides (ƩDDTs) was set at 10 μg/kg bw (Bouwman et al., 2006), and TDI for sum of chlordanes (ƩChlDs) was set at 500 ng/kg bw (EFSA European Food Safety Authority, 2007).

### 2.9 Ethical considerations

This study follows the ethical principles for research on human biological samples stated by the Helsinki Declaration. The study received approval from the Norwegian Regional Committees for Medical and Health Research Ethics (REC South‐East C), under the reference number 26762. All participants gave informed consent and were informed that additional analyses might be conducted on the collected breastmilk samples.

## 3 RESULTS

Of the 120 included women in the current study, 110 (91.7%) completed the general demographic characteristics questionnaire. The average maternal age was 30.9 years, and the majority (90.9%) of the participants were born in Norway. Only three participants reported currently smoking cigarettes. The ages of the children ranged from 0 to 22 months, with a median age of 4.5 months, and 57.4% were male (Table 1).

**Table 1:** General demographic characteristics of mothers and infants, presented as mean with standard deviation (SD) or number with percent (*N* = 110).

	*n*	Mean (SD) or %
Maternal age (years)	110	30.9 (4.4)
BMI (kg/m^2^) pre‐pregnancy	109	24.8 (4.9)
Maternal parity		
0	45	41.3
1	46	42.2
≥2	18	16.5
Country of birth, Norway	100	90.9
Smoking status, No smoking	107	97.3
Child's sex		
Female	46	42.6
Male	62	57.4
Child's age when sample was collected (Months)		
0–1	29	26.4
2–3	16	14.6
4–5	28	25.5
6–7	16	15.5
8–11	14	12.7
12≥	6	5.5

PCBs were detected in 100% of the samples, while ChlDs and DDTs were detected in 98.3% of the samples (Table 2). Concentrations of Ʃ_13_PCBs ranged between 6.6 and 81.0 ng/g lw, with a median of 26.9 ng/g lw. For ƩDDTs, the concentrations varied from not detected (ND) to 2059.4 ng/g lw, with a median of 17.4 ng/g lw. Similarly, for ƩChlDs, the concentrations ranged from ND to 33.8 ng/g lw, with a median of 3.0 ng/g lw. HCHs and BDEs were detected in 66.7% and 72.5% of the samples, respectively, with concentrations ranging from ND to 11.9 ng/g lw and ND to 5.4 ng/g lw, and a median of 3.0 ng/g lw and 0.6 ng/g lw, respectively. HBCDD was found in 30% of the samples, with concentrations ranging from ND to 7.0 ng/g lw and a median of 0.4 ng/g lw. The lowest detection percentage was for Mirex, which was identified in 29.2% of the samples, with concentrations ranging from ND to 2.6 ng/g lw and a median of 0.2 ng/g lw.

**Table 2:** Median concentration of 35 different POPs detected in *N* = 120 breast milk samples in Innlandet County, Norway. Presented as wet weight (ww, ng/g milk) and lipid weight (lw, ng/g fat).

	*n* a (%)	50p ww	5p–95p ww	50p lw	5p–95p lw	LOD
α‐HCH	7 (5.8)	0.003	0.002–0.005	0.062	0.041–0.142	0.003
β‐HCH	77 (64.2)	0.045	0.020–0.813	1.041	0.547–2.851	0.002
γ‐HCH	15 (12.5)	0.010	0.004–0.158	0.230	0.081–2.467	0.002
ƩHCHs	80 (66.7)	0.047	0.016–0.813	1.110	0.479–3.063	.
*p*,*p*′‐DDE	117 (97.5)	0.652	0.139–6.156	16.919	6.364–46.786	0.046
*o*,*p*’‐DDD	0 (0)	<LOD	<LOD	<LOD	<LOD	0.023
*p*,*p*’‐DDD	0 (0)	<LOD	<LOD	<LOD	<LOD	0.021
*o*,*p*’‐DDT	0 (0)	<LOD	<LOD	<LOD	<LOD	0.019
*p*,*p*’‐DDT	30 (25.0)	0.047	0.027–3.383	1.095	0.650–2.340	0.025
ƩDDTs	118 (98.3)	0.655	0.139–6.156	17.427	6.364–46.786	.
Oxychlordane	115 (95.8)	0.033	0.011–0.148	1.041	0.438–2.327	0.002
*trans*‐Chlordane	0 (0)	<LOD	<LOD	<LOD	<LOD	0.002
*cis*‐Chlordane	8 (6.7)	0.004	0.002–0.006	0.080	0.042–0.096	0.002
*trans*‐Nonachlor	117 (97.5)	0.060	0.011–0.294	1.506	0.543–3.743	0.002
*cis*‐Nonachlor	111 (92.5)	0.014	0.003–0.067	0.367	0.090–1.011	0.002
ƩChlDs	118 (98.3)	0.109	0.020–0.474	2.953	1.057–6.231	.
Mirex	35 (29.2)	0.009	0.004–0.074	0.243	0.125–0.915	0.002
PCB‐28	84 (70.0)	0.021	0.006–1.751	0.555	0.167–4.697	0.004
PCB‐31	8 (6.7)	0.013	0.011–0.023	0.534	0.255–1.364	0.004
PCB‐101	43 (35.8)	0.053	0.027–0.109	1.351	0.790–2.495	0.025
PCB‐105	114 (95.0)	0.013	0.003–0.047	0.355	0.155–0.826	0.003
PCB‐114	79 (65.8)	0.004	0.003–0.016	0.120	0.059–0.277	0.002
PCB‐118	120 (100)	0.054	0.015–0.161	1.560	0.692–2.984	0.003
PCB‐138	120 (100)	0.188	0.049–0.605	5.518	2.269–11.101	0.004
PCB‐153	120 (100)	0.312	0.090–1.095	9.286	3.958–19.249	0.005
PCB‐156	120 (100)	0.033	0.007–0.041	0.981	0.364–2.136	0.006
PCB‐157	113 (94.2)	0.009	0.003–0.036	0.289	0.123–0.520	0.007
PCB‐170	113 (94.2)	0.063	0.007–0.301	2.062	0.207–4.464	0.008
PCB‐180	120 (100)	0.149	0.046–0.660	4.697	1.790–10.616	0.009
PCB‐189	62 (51.7)	0.004	0.002–0.011	0.088	0.045–0.183	0.010
ƩPCBs	120 (100)	0.934	0.255–2.920	26.915	11.120–52.925	.
BDE‐28	2 (1.7)	0.009	0.007–0.011	0.223	0.205–0.242	0.006
BDE‐47	41 (34.2)	0.013	0.008–0.079	0.337	0.123–1.842	0.008
BDE‐99	3 (2.5)	0.026	0.022–0.034	0.598	0.576–0.766	0.021
BDE‐100	7 (5.8)	0.017	0.011–0.062	0.360	0.323–1.148	0.009
BDE‐153	76 (63.3)	0.017	0.009–0.056	0.462	0.180–1.283	0.009
BDE‐154	6 (5.0)	0.008	0.006–0.009	0.251	0.077–0.469	0.006
BDE‐183	1 (0.8)	0.016	0.016–0.016	0.366	0.366–0.366	0.025
ƩBDEs	87 (72.5)	0.023	0.010–0.092	0.593	0.221–2.709	.
HBCDD	36 (30.0)	0.071	0.056–0.107	1.836	0.840–5.490	0.056

Abbreviations: BDE, brominated diphenyl ether; HBCDD, hexabromocyclododecane; HCH, hexachlorocyclohexane; LOD, Limits of detection (ng/g breast milk); lw, lipid weight (ng/g of fat); *p*,*p*′‐DDD, dichlorodiphenyl‐dichloroethane; *p*,*p*′‐DDE, dichlorodiphenyldichloroethylene; *p*,*p*′‐DDT, dichlorodiphenyltrichloroethane; PCB, polychlorinated biphenyl; ww, wet weight (ng/g breast milk).

^a^ Number of detected samples.

A positive association between maternal age and ƩChlDs lw (RM = 1.04) was observed, reflecting a 4% increase in concentration per year (95% CI 1.00–1.08) (Table 3). A similar association was also observed between maternal age and Ʃ_13_PCBs lw (RM = 1.03), showing a 3.1% increase per additional year of maternal age (95% 1.01–1.05). Furthermore, a negative association was found between infant age in months and ƩDDTs and ƩChlDs levels. For ƩDDTs, the estimated association was an RM of 0.96, suggesting a 3.8% drop in concentration per month increase in infant age (95% CI 0.93–0.99). For ƩChlDs lw the RM was 0.92 (95% CI 0.89–0.96), indicating a 7.8% decrease in concentration per month increase in infant age. Parity was only associated with the outcome ƩChlDs lw with an RM of 0.71 (95% CI 0.52–0.98), indicating a 28.7% reduction in concentration between the mothers' first and second child. Conversely, para 2 was associated with a drop in concentration of ƩDDTs lw (RM = 0.60, 95% CI 0.39–0.93) when compared to para 0.

**Table 3:** Estimates from multiple regression analyses using negative binomial regression models displaying percentage increase or decrease in concentration.

	**Ʃ**DDTs lw		ƩChlDs lw		ƩPCBs lw	
	RM	95% CI	RM	95% CI	RM	95% CI
Woman's age (years)	**1.03**	**1.00–1.06**	**1.04**	**1.00–1.08**	**1.03**	**1.01–1.05**
Parity (Reference is para 0)						
1	0.93	0.70–1.24	**0.71**	**0.52–0.98**	0.88	0.74–1.05
2	**0.60**	**0.39–0.93**	0.65	0.41–1.03	0.79	0.61–1.03
3	0.70	0.33–1.47	0.60	0.25–1.41	0.77	0.48–1.23
Pre‐pregnancy BMI (kg/m^2^)	1.01	0.99–1.04	1.00	0.97–1.03	0.98	0.98–1.01
Infant age (months)	**0.96**	**0.93–0.99**	**0.92**	**0.89–0.96**	0.96	0.96–1.00

*Note*: ƩHCHs = sum concentration of α‐HCH (hexachlorocyclohexane), β‐HCH, γ‐HCH; ƩDDTs = sum concentration of *p,p*′‐DDE (dichlorodiphenyldichloroethylene), *o,p*′‐DDD (dichlorodiphenyl‐dichloroethane), *p,p*′‐DDD, *o,p*′‐DDT (dichlorodiphenyltrichloroethane) and *p,p*′‐DDT; ƩChlDs **=** Oxychlordane, *trans‐*chlordane, *cis‐*chlordane, *trans*‐nonachlor and *cis*‐nonachlor; ƩPCBs(polychlorinated biphenyls) **=** PCB‐28, −31, −2, −101, −105, −114, −118, −138, −153, −156, −157, −170, −180 and −189. Bold values are *p* < 0.05.

Abbreviations: BMI, body mass index; CI, confidence interval; lw, lipid weight (ng/g fat); RM, ratios of means.

No associations were observed between the outcomes ƩHCHs lw, Mirex lw, ƩBDEs lw, and HBCDD lw and the independent variables maternal age, parity, pre‐pregnancy BMI, or infant age. Moreover, no association was observed between the POPs categories and pre‐pregnancy BMI.

**Figure 2:**
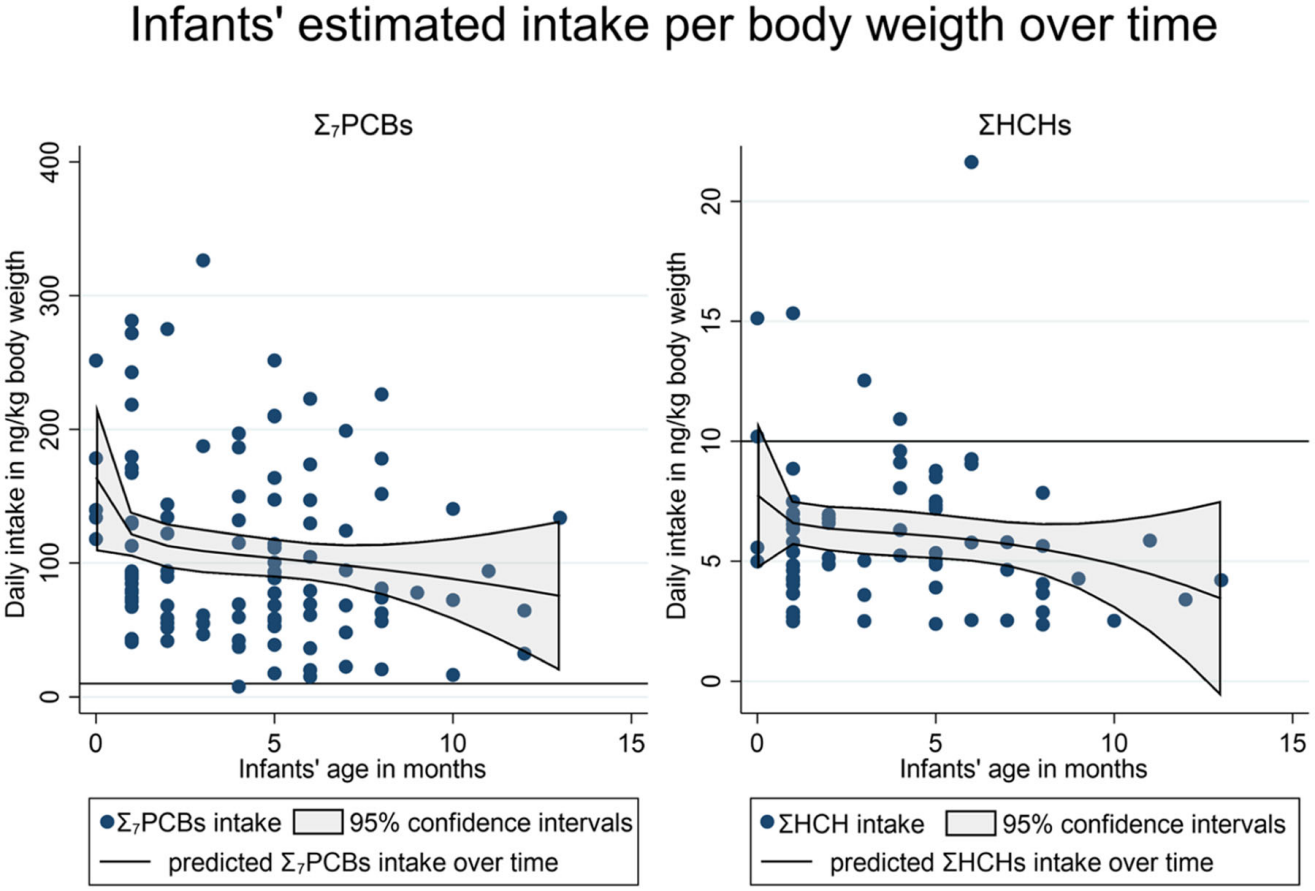
Estimated daily intake over time; dots: estimated total daily intake per sample; declining line: predicted intake over time with 95% confidence intervals; horizontal line: health‐based guides value of 10 ng/kg body weight Ʃ_7_PCBs (polychlorinated biphenyl) = PCB‐28, ‐52, ‐101, ‐118, ‐138, ‐153, and ‐180 (Skåre et al., 2008) and ƩHCHs (hexachlorocyclohexane = α‐HCH, β‐HCH, and γ‐HCH) (WHO, 2003).

Based on the evaluations of estimated daily intake per body weight, 6.4% (7/110) of the included infants surpassed the health‐based guidance values for ƩHCHs. Notably, 99.1% (109/110) of the infants exceeded the health‐based guidance values for Ʃ_7_PCBs (Figure 2).

None of the infants had intakes of ƩDDTs and ƩChlDs above the health‐based guidance values.

## 4 DISCUSSION

In this cross‐sectional study, PCBs, DDTs, and ChlDs were detected in 100%, 98.3% and 98,3% of the included breastmilk samples, respectively. The concentrations of the 35 different POPs measured varied and the highest median concentration of 26.9 ng/g lw was found for the ƩPCBs. The highest overall concentration of 2059.4 ng/g lw was found for ƩDDTs. Maternal age, parity, pre‐pregnancy BMI, and infant age were significant determinants for different POPs concentrations. There was a positive association between maternal age, ƩChlDs lw, and ƩPCBs lw, while infant age showed negative associations with ƩDDTs lw, and ƩChlDs lw. Parity demonstrated different associations with POPs concentrations. Based on estimated daily intake per body weight, about 6% of the infants exceeded health‐based guidance values for ƩHCHs, while 99% exceeded the values for ƩPCBs. None of the infants exceeded the health‐based guidance values for ƩDDTs and ƩChlDs.

The existing body of literature consistently identifies three major predictors for higher levels of POPs in females: older age, BMI and nulliparity (Wesselink et al., 2019). Furthermore, women who breastfeed for a longer duration generally exhibit lower POPs concentrations (Wesselink et al., 2019). Given this context, it is not surprising that our data showed associations between mothers' age, parity, and infants' age and the concentration of POPs in breastmilk. However, the associations are relatively few. This limited finding could be attributed to the low sample size, coupled with the low concentrations and low detection percentages for the different POPs. Interestingly, the present study found no significant association between POPs concentrations and BMI. This could be related to the previous reasons mentioned, as well as to the fact that the mean BMI of our study population was 24.8 (SD 4.9), indicating a limited variation in BMI among the participants.

A median concentration of 26.9 ng/g lw for Σ_13_PCBs in breastmilk was observed in the present study, which is notably lower than previously reported values in Norway (Polder et al., 2009). However, 99.1% of infants still consume more Ʃ_7_PCBs than the recommended health‐based values. There have not been studies on POPs in breastmilk in Norway since the mid‐2000s (Knutsenet al., 2010). However, serum samples from pregnant women, participating in the Northern Norway Mother‐and‐Child Contaminant Cohort (MISA study), reveal a marked decline in dioxins, PCBs, and pesticides from 2007 to 2009 to 2019 (Xu et al., 2022). This decline may be attributed to the prohibition of POPs in Europe and USA from the 1970s onward, and international regulation via the Stockholm Convention. Other measures initiated in the late 1990s, such as soil remediation and sediment removal from polluted locations may also have contributed the decline (Fjeld et al., 2012). The decreasing trend is not confined to Norway; both the World Health Organization and the United Nations Environment Programme (WHO/UNEP) have reported global reductions in POPs concentrations in human breastmilk (Malisch, 2017). According to a 2013 report, there has been a consistent decline in various POPs worldwide (WHO/UNEP, 2013). Despite this downward trajectory, the present levels of ΣPCBs detected in breastmilk remain a health concern (WHO/UNEP, 2013). Thereby requiring a risk‐benefit assessment concerning breastfeeding. However, both WHO/UNEP and the Norwegian Science Committee for Food and Environment determined in 2013 that the advantages of breastfeeding outweigh the potential risks posed by environmental pollutants (Meltzer et al., 2013; WHO/UNEP, 2013). This was further confirmed by the Committee on Nutrition of the French Society of Paediatrics in 2021, specifying that while the presence of POPs in breastmilk poses potential health consequences, there is an overwhelming scientific consensus that breastfeeding remains the optimal food source for infants (Lapillonne et al., 2021). Given the continued decline in these concentrations, we do not believe that the potential health risk has increased.

Further examination of the limited existing literature on the half‐lives (Drobná et al., 2011; Grandjean et al., 2008; Ogura, 2004) of different PCBs reveals interesting patterns. For example, PCB‐105 and PCB‐28, which have half‐lives of 5–6 years, are detected in 90% and 70% of samples, respectively, with median concentrations of less than 1 ng/g lw. In contrast, PCB‐138 and PCB‐180, which have longer half‐lives of 10‐12 years, are detected in 100% of the breastmilk samples, with median concentrations of 5.5 and 4.7 ng/g lw, respectively. Surprisingly, PCB‐170, which has an even longer half‐life of 15.5 years, is detected in 94.2% of the samples, with a median concentration of 2.1 ng/g lw. Despite its presence in fewer samples and at lower concentrations, the concentration of PCB‐170 has decreased 4.5‐fold since the early 2000s. Meanwhile, the concentration of PCB‐180 has declined by 11.7‐fold over the same time period (Polder et al., 2008).

In the present study, large individual variations in contaminant levels were evident, particularly in the concentrations of ΣDDTs. These findings are consistent with prior Norwegian studies (Forns et al., 2016; Lenters et al., 2019), yet a nearly three‐fold decrease is observed when compared to the 1999–2008 HUMIS cohort (Lenters et al., 2019). According to the 2013 WHO/UNEP report, high concentrations of ΣDDTs were notably prevalent in tropical and subtropical regions, often attributed to malaria control measures in these areas (Bouwman et al., 2011; Tren & Roberts, 2010; Wassie et al., 2012; WHO/UNEP, 2013). While specific data on ethnicity are lacking in our cohort, elevated ΣDDTs levels might be associated with immigrants from these regions, corroborating previous studies that reported significant disparities in DDTs levels between ethnic Norwegian and non‐Norwegian women (Lenters et al., 2019). This hypothesis could explain the discrepancy between the 95th and 100th percentile values in this study (95th percentile = 46.8 ng/g lw, 100th percentile = 2059.4 ng/g lw). Additionally, a 2013 WHO/UNEP survey highlighted lower concentrations of other chlorinated pesticides, such as Mirex, present in only 29.2% of these samples (WHO/UNEP, 2013).

It is crucial to acknowledge the elevated levels of BDEs historically detected in Lake Mjøsa's fish population, attributed to industrial discharges from a local source at Lillehammer, situated at the lake's northernmost edge (Berg et al., 2013). The concentrations of BDE and HBCDD reported in that study rank among the highest concentrations ever detected in biological matrices. These elevated levels extend to human subjects consuming fish from this lake, further corroborating the potential for elevated BDE concentrations in our cohort (Thomsen et al., 2008). Nevertheless, the study indicates a substantial reduction in Ʃ_7_BDE concentrations, a 3.6‐fold decrease from 2.2 ng/g lw in the 1999–2008 HUMIS study to 0.6 ng/g lw in the present cohort (Lenters et al., 2019). HBCDD was identified in 30% of our samples, reinforcing the reduction of these contaminants. It is worth noting that there is an absence of a tolerable daily intake (TDI) value for ƩBDEs from the Norwegian Science Committee for Food and Environment, although the need for more comprehensive research was underscored in a 2005 report (Skåre et al., 2005). The Netherlands National Institute for Public Health and the Environment has proposed a TDI value of 2.3 pg/kg bw per day for BDE‐99 (de Winter‐Sorkina et al., 2006). Intriguingly, based on this TDI criterion, a substantial 72.7% of infants in our study would exceed this health‐based guideline for Ʃ_7_BDEs, necessitating further enquiry into potential health implications.

This study provides contemporary data on the levels of POPs in breastmilk, noting a significant decline in concentrations over time. It confirms determinants affecting these concentrations in previous studies, such as maternal age and infant age, while distinctively finding no significant association with pre‐pregnancy BMI. The study lengthens the usual time studied for POPs intake through breastfeeding to a year, offering important data that could affect future guidelines in Norway. However, it also shows that more research is needed, especially with a larger and more varied group of participants. Other factors like diet, lifestyle, and local environmental conditions also need to be taken into consideration. The study also points out the need for more research on the long‐term health effects of these pollutants and on pollutants like ΣBDEs, for which there are currently no set guidelines in Norway.

### 4.1 Strengths and limitations

One of the major strengths of this study is that it provides a year‐long estimate curve for POPs intake via breastfeeding, offering valuable data that could influence future breastfeeding guidelines. Additionally, the analytical quality was reliable; the relative recovery in percent was in the 90th percentile for the majority of the components analysed, with only six components exhibiting relative recovery in the 80th percentile. Despite these strengths, the study has some limitations. This study was primarily designed to describe the iodine status and iodine intake in the mothers and their infants, and the questionnaires therefore lacked some relevant questions for a broader analysis of POPs. Additionally, the biological samples were mainly collected to determine the concentration of iodine. Consequently, available data on breastmilk intake volume and each infant's weight were not collected at the same time, resulting in less precise intake estimations. Another limitation is the deviation of our study design from the WHO/UNEP human milk survey guidelines, where the breastmilk samples where not collected at the same timepoint for all participants.

## 5 CONCLUSION

PCBs were detected in 100%, and DDTs and ChlDs in 98.3% of the breastmilk samples analysed in the present study, but in lower concentrations than previous studies from Norway. Despite the decline in concentrations, most breastfeeding infants still consume Σ_7_PCBs that exceed the tolerable daily intake. Additionally, about 6% of these infants exceeded the tolerable daily intake of ΣHCHs. When comparing the intake of BDEs with the TDI proposed by Netherlands National Institute for Public Health, 72.7% of infants in our study would exceed this proposed health‐based guideline for Ʃ_7_BDEs. It should be noted that both WHO/UNEP and the Norwegian Science Committee for Food and Environment concluded in 2013 that the benefits of breastmilk for a child surpass the potential risks posed by these environmental pollutants. Given that breastmilk is a critical source of nutrition for infants, it is necessary to continue monitoring and researching POPs in breastmilk to better understand their long‐term health implications.

## AUTHOR CONTRIBUTIONS

Kristina R. Nermo contributed to conceptualization, methodology, formal analysis, investigation, data curation, writing original draft and visualization. Jan L. Lyche contributed to conceptualization, methodology, validation, resources and writing original draft. Gabrielle Haddad‐Weiser contributed to validation, investigation, resources, data curation and writing original draft. Tonje E. Aarsland, Siri Kaldenbach and Beate Solvik contributed to investigation and data curation. Anuschka Polder contributed to validation and writing original draft. Tor A. Strand contributed to conceptualization, methodology, formal analysis, writing original draft, supervision, project administration and funding acquisition. Kjersti S. Bakken contributed to conceptualization, methodology, formal analysis, investigation, data curation, writing original draft, visualization, supervision and project administration.

## CONFLICT OF INTEREST STATEMENT

The authors declare no conflict of interest.

## Data Availability

The data that support the findings of this study are available on request from the corresponding author. The data are not publicly available due to privacy or ethical restrictions.
